# Distinguishing Benign from Malignant Small Enhancing Breast Lesions Using Radiomics

**DOI:** 10.3390/diagnostics16172818

**Published:** 2026-09-02

**Authors:** Parlyn Hatch, Christopher Louviere, Mutlu Mete, Oswaldo A. Guevara Tirado, Ruben G. Ortiz Cordero, Hector Diaz De Villegas, Kazim Z. Gumus

**Affiliations:** 1 Department of Radiology, College of Medicine-Jacksonville, University of Florida, 655 West 8th Street, C506, Jacksonville, FL 32209, USA; parlyn.hatch@jax.ufl.edu (P.H.);; 2 Department of Radiology, University of Florida Health Jacksonville Physicians Inc., 655 West 8th Street, C506, Jacksonville, FL 32209, USA; 3 Department of Information Science, University of North Texas, 620 Central Avenue, Denton, TX 76203, USA

**Keywords:** radiomics, breast MRI, breast lesions, machine learning, multilayer perceptron

## Abstract

**Background/Objectives:** Small enhancing lesions (often termed foci if lesion ≤ 5 mm) observed on breast magnetic resonance imaging (MRI) pose a persistent challenge. Their clinical relevance and the most effective management strategy remain unclear, often leading to unnecessary biopsies. We sought to uncover whether quantitative radiomic features of these lesions could reliably discriminate malignant ones from benign ones. **Methods:** In this single-center retrospective study (2015–2024), we analyzed 31 contrast-enhancing breast lesions with a maximum diameter ≤ 10 mm measured on a single axial MRI slice from 30 patients who underwent 1.5-T or 3.0-T breast MRI followed by histologic confirmation (10 malignant, 21 benign). Lesions were manually delineated, and 105 radiomic variables were extracted from early-phase dynamic contrast-enhanced (DCE) subtraction images. Feature importance was quantified with a Random Forest model; iterative top-k pruning found the highest-performing variables. A multilayer perceptron (MLP) classifier was trained and validated using a leave-one-subject-out cross-validation (LOSO-CV) scheme, with the sensitivity, specificity, accuracy, and AUC as the primary performance metric. **Results:** Of the 105 extracted features, 44 carried predictive information (feature importance score ≥ 0.20). Progressive feature reduction yielded an optimal subset of nine radiomic features (six texture-based and three shape-based). Texture-derived features predominated among the most informative variables. The MLP achieved a sensitivity of 0.80, specificity of 0.89, accuracy of 0.86, and AUC of 0.82. **Conclusions:** In this exploratory study, a preliminary nine-feature radiomic signature extracted from early-phase DCE-subtraction images has the potential to distinguish benign from malignant small breast lesions when used with an MLP model. Prospective validation is needed before the model can influence clinical decision-making.

## 1. Introduction

Breast cancer is among the leading causes of cancer-related death in women, with U.S. incidence rising 1% between 2020 and 2021 according to the Centers for Disease Control and Prevention [1]. To standardize reporting and improve communication among healthcare professionals, the American College of Radiology (ACR) developed the Breast Imaging Reporting and Data System (BI-RADS) [2]. Since the implementation of BI-RADS in 1993, magnetic resonance imaging (MRI) has become a vital imaging tool for breast cancer diagnosis, providing a diagnostic accuracy of 86.9% [3,4]. However, studies report false-negative breast MRI results of approximately 4–7% in preoperative MRI-pathology correlation studies [5,6]. Although biopsy remains the gold-standard for definitive diagnosis of breast cancer, a unique advantage of MRI is that it allows for the detection of small enhancing lesions, including those previously defined by ACR BI-RADS as foci (≤5 mm) [7,8,9,10]. Although these small lesions are often associated with increased hormonal stimulation, they can also represent a developing malignancy [9]. Because malignancy rates of small lesions are variable (ranging from 0.6 to 23%), their clinical significance and optimal management remain unclear [9]. Early detection of malignant small lesions is of particular interest in high-risk women, where timely identification could lead to meaningful improvements in management and outcomes.

Radiomics, the extraction of mathematical features from medical images through a data characterization algorithm, could help address the uncertainty surrounding the interpretation of small enhancing lesions [11,12,13]. Selected features can be reduced to the most predictive and used to train a machine learning (ML) model to classify breast cancer lesions and estimate the probability of malignancy [7,13,14,15,16,17].

In this preliminary study, we explored radiomic features of breast lesions ≤ 1 cm in size from contrast-enhanced T1-weighted images. We hypothesized that an ML model could be trained to classify small enhancing breast lesions using the selected features and accurately predict malignancy.

## 2. Material and Methods

### 2.1. Patient Selection

**Figure 1:**
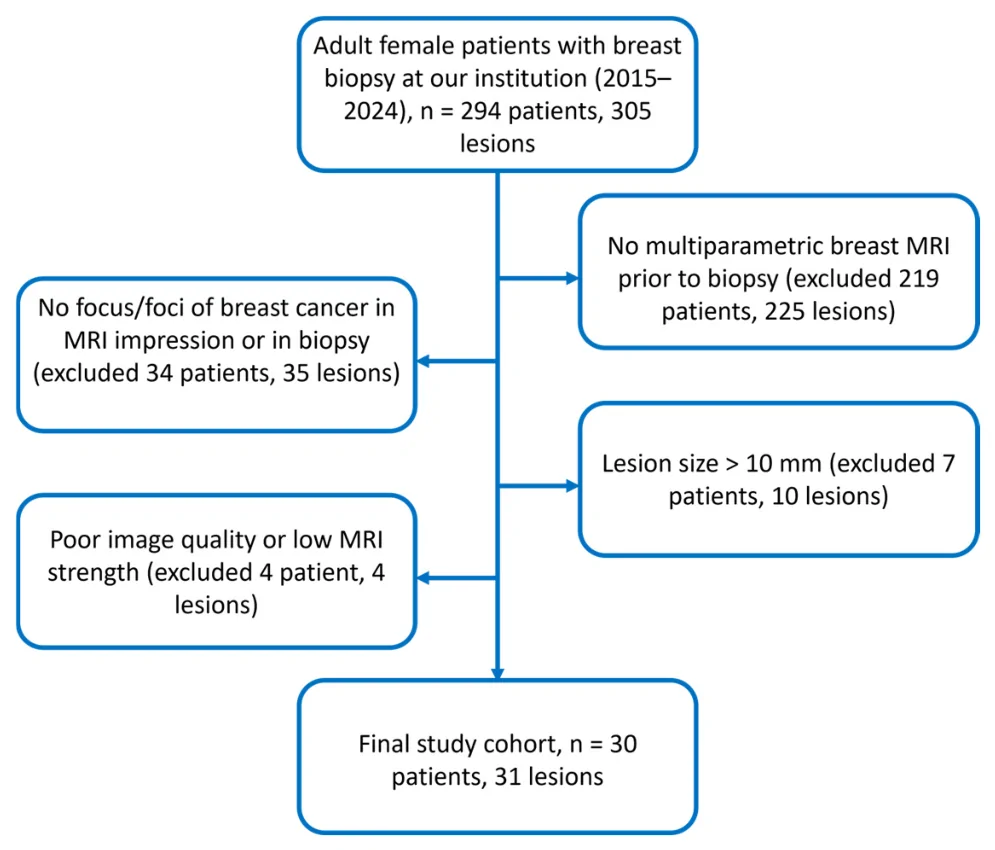
Flowchart of patient selection and study cohort derivation. The diagram summarizes the identification, screening, inclusion, and exclusion of patients from the initial dataset retrieval to the final study cohort used for analysis. Reasons for exclusion at each stage are provided where applicable.

We obtained Institutional Review Board (IRB) approval for this retrospective study (IRB approval number: IRB202401789; date of approval: 6 February 2025). The requirement for written informed consent was waived by the IRB given the retrospective design of the study. We reviewed the medical records of adult female patients who underwent multiparametric breast MRI using a 1.5-T or 3.0T scanner at our institution and subsequently had a breast biopsy from 2015 through 2024. A total of 30 female patients with 31 enhancing breast lesions were selected based on the MRI reports and pathology results. To ensure all lesions would be ≤10 mm in size, patients were included if their “impressions” stated that a breast cancer focus/foci was visualized. A flowchart summarizing the cohort selection process is presented in Figure 1. Lesion size was measured in millimeters (mm) utilizing the segmented area in the imaging data and ITK-SNAP software (version 3.8.0). A two-sample *t*-test was performed to compare the size measurements.

### 2.2. MRI Protocol

Images were acquired on a 1.5-T scanner (Siemens Espree) or 3-T scanners (Siemens TrioTim, Siemens Vida, and Siemens Skyra, Siemens, Erlangen, Germany). Patients were imaged in the prone position with a dedicated multi-channel phased-array breast coil per institutional standards. The imaging protocol included axial T1-weighted, axial T2-STIR, and an axial T1-weighted dynamic contrast-enhanced (DCE) images with six phases: one pre-contrast and five post-contrasts. Gadoteric acid (Dotarem^®^, Guerbet, Princeton, NJ, USA) was used as a contrast agent at a dose of 0.2 mL/kg with an injection rate of 2 mL/s. DCE scan parameters obtained for both fields are shown in Table 1.

### 2.3. Segmentation

DCE images were sent to DynaCAD (version 5.0) (Invivo, Gainesville, FL, USA) software, where pre-contrast images were subtracted from post-contrast images to create subtraction images. The first subtraction image (first-phase post-contrast image minus pre-contrast image) was used for radiomic analysis. The DICOM files were then de-identified, extracted from the Visage Picture Archive and Communication System (PACS) software, imported into ITK-SNAP, and converted to Neuroimaging Informatics Technology Initiative (NIfTI) format for segmentation [18]. Lesions were manually segmented slice-by-slice by two trained radiology research fellows in consensus and confirmed by an MR physicist and a breast radiologist. The resulting segmentation masks and original image datasets were then used to extract radiomic features and train a machine learning (ML) algorithm.

### 2.4. Experimental Design

The unit of analysis in this study was the image of the lesion. One patient contributed two lesions, one benign and one malignant, which were entered as separate samples. Because classification relied exclusively on radiomic features computed from the voxels of each individual lesion, and no patient-level or demographic variables were included in the feature set; treating these two lesions as independent observations does not transfer shared patient information across folds. In the context of lesion classification, the presence of breast cancer was treated as a positive class. Classification models were trained and validated using a leave-one-subject-out (LOSO) cross-validation strategy, in which a lesion is excluded from the dataset in each iteration and used as the test set while the model is trained on the data of all remaining lesions. LOSO was chosen due to its advantage of minimizing subject-specific bias, ensuring that the classifier’s performance generalizes well across lesions. Each experiment was repeated 30 times using different random seeds. The primary evaluation objective is to maximize the GeoMean of sensitivity (true positive rate) and specificity (true negative rate), given as $Sen=\;\frac{\mathrm{TP}}{\mathrm{TP}\;+\;\mathrm{FN}}$, $Spe\;=\;\frac{\mathrm{TN}}{\mathrm{TN}\;+\;\mathrm{FP}}$, and $GeoMean\;=\;\sqrt{Sen*Spe}$. Geometric mean is useful for imbalanced datasets, as it ensures both sensitivity and specificity are high, preventing a model from simply maximizing accuracy by predicting the majority class.

### 2.5. Feature Selection

Using the PyRadiomics library (version 3.1.0) [19], first, we normalized the signal intensities on the DCE images to eliminate scan-to-scan variations. In-plane resampling was performed on the images (0.6 × 0.6 mm) to homogenize image processing findings. Then, we extracted 105 radiomic features from each 3D lesion per imaging sequence. Radiomics features were of the following feature classes: first order (*n* = 18), shape (*n* = 14), and texture (*n* = 73) including Grey Level Co-occurrence Matrix (GLCM, *n* = 22), Grey Level Size Zone Matrix (GLSZM, *n* = 16), Grey Level Run Length Matrix (GLRLM, *n* = 16), Grey Level Dependence Matrix (GLDM, *n* = 14), and Neighboring Gray Tone Difference Matrix (NGTDM, *n* = 5). Most of the features are in line with the Imaging Biomarker Standardization Initiative (IBSI) [20].

Feature selection and ranking are critical steps in high-dimensional radiomic feature classification. Although a comprehensive feature set can encapsulate rich spatial dynamics, it also increases the risk of overfitting, redundancy, and computational inefficiency. Therefore, identifying a subset of the most informative and discriminative features is essential for enhancing model performance and interpretability.

We employed the Feature Importance Score (FIS) of the Random Forest (RF) Algorithm to rank all 105 features. The RF is an ensemble of decision trees that aggregates their predictions and quantifies feature importance from the reduction in node impurity attributable to each feature [21]. Node impurity is measured by the Gini impurity, defined for a node t as $G(t)=1-\sum_{i=1}^{C}p_{i}^{2}$, where *p_i_* is the proportion of samples of class *i* among the *C* classes at that node. It equals zero for a pure node containing a single class and increases as the classes become more evenly mixed, and it can be read as the probability of misclassifying a randomly chosen sample labeled according to the node’s class distribution [22]. The importance of a feature is the total Gini impurity reduction across all splits on that feature, weighted by the sample count at each node and averaged over the trees, as implemented in Scikit-learn [23]. The FIS is the Gini-based mean decrease in impurity, averaged over the 64 trees of each Random Forest, and normalized to sum to one across the 105 features per run. The score was computed over 16 random seeds and aggregated by summation across runs, so the reported values lie on a 0 to 16 scale, with the most informative feature reaching 1.81; the corresponding mean per run values are obtained by dividing by 16. The 0.20 cutoff is a descriptive label for features carrying non-negligible information and was not used to select the classification feature set, which was determined by the top-k Geometric Mean sweep.

We implemented a top-k iterative pruning strategy, a form of recursive feature elimination in which the features with the lowest importance scores are incrementally removed and the classifier is re-evaluated at each step [24,25], using the Scikit-learn library [23].

### 2.6. Classification Algorithm: Multilayer Perceptron

Our classification approach used a Multilayer Perceptron (MLP) neural network trained by the backpropagation algorithm [26,27]. The network was optimized with the Adam optimizer [28] and implemented in PyTorch (Version 2.9) [29]. Data preparation involved subjecting all input features to standardization (Z-score normalization). This preprocessing step is vital for significantly enhancing the efficiency and stability of the training process of the neural network. By transforming the features to have a zero mean and unit variance, we ensured that the MLP algorithm encountered a more spherical and uniform error surface. This ultimately leads to faster convergence, as the optimizer is prevented from oscillating back and forth across uneven scales. Furthermore, scaling the inputs mitigates the significant threat of gradient vanishing or exploding problems that can arise from highly variable input magnitudes.

The MLP was structured with an input layer, output layer, and a sequence of four internal hidden layers. The specific size of these hidden layers was not fixed but dynamically configured based on the total number of input features, *N*. The MLP architecture was configured according to the number of input features *N*. For *N* greater than 64, four hidden layers of 128, 64, 32, and 16 neurons were used; for *N* less than or equal to 64, three hidden layers of 64, 32, and 16 neurons were used. As the final model was trained on nine selected features, it used the three hidden layer configuration (64, 32, 16).

The MLP was trained with the binary cross-entropy with logits loss, weighting the positive (malignant) class by 1.7 to address class imbalance, using the Adam optimizer (learning rate 0.001, batch size 2). Each fold was trained for up to 30 epochs with early stopping (patience 7 epochs, minimum loss improvement 0.0001). Hidden layers used ReLU activation, and the output layer produced a logit passed through a sigmoid, with a fixed decision threshold of 0.50. Weights were initialized using the Xavier uniform method with zero biases. No dropout or weight decay was applied; early stopping functioned as the sole form of regularization. The positive (malignant) class was assigned a fixed weight of 1.7 in the loss function to address class imbalance. This value was chosen a priori to approximate, while remaining below, the inverse class frequency of the cohort (21 benign versus 10 malignant lesions) and was held constant across all folds. Because the weight was not estimated from the data, it does not differ between folds and introduces no fold-dependent leakage.

**Figure 2:**
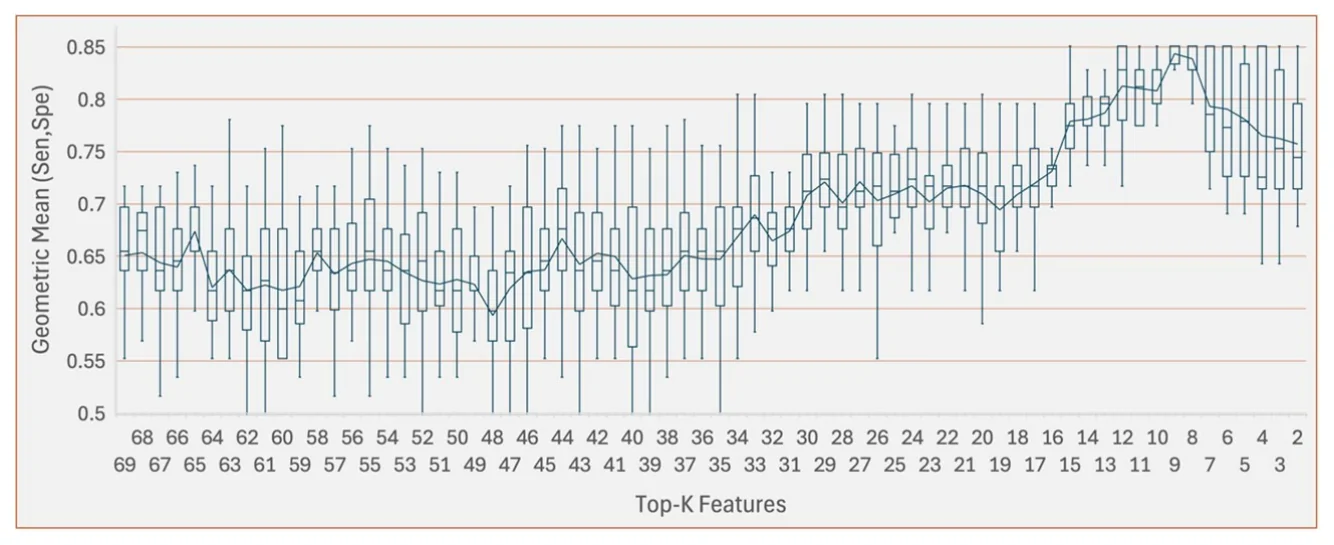
Box-and-whisker plot showing cross-validated distribution of Geometric Mean by Ranked Feature Sets (Sen: Sensitivity, Spe: Specificity).

The value of k in the Feature Selection algorithm was iterated from 105 to 2 by 1. For each set of k features, the models to be tested were trained using leave-one-subject-out cross-validation (LOSO-CV), and the geometric mean of each trained model was calculated. The set of features that yielded the highest GeoMean was identified as the best model. Although k was iterated from 105 down to 2, Figure 2 displays the GeoMean distribution across feature set sizes from k = 69 down to k = 2 for visualization clarity, as feature sets beyond k = 69 contributed negligibly to discriminative performance. The models were implemented using Python version 3.9.5, SciKitLearn version 1.4.0, and XGBoost version 1.5.1 [30]. The network architecture, optimizer, learning rate, batch size, weight initialization, and class weight were fixed a priori and were not tuned by grid search or nested cross-validation; the number of training epochs was governed by early stopping. The only empirically selected setting was the number of retained features, k, which was chosen to maximize the cross-validated GeoMean. This selection was not nested within the outer cross-validation folds, and a nested procedure that confines feature and hyperparameter selection to each training fold is required for unbiased estimation in future work.

### 2.7. Statistical Analysis

The confusion matrix was used to determine instances in a predicted class. The accuracy, sensitivity, and specificity values for the radiomics classifier were calculated. The AUC of the receiver operating characteristic curve for malignant-versus-benign was calculated.

### 2.8. Use of Generative Artificial Intelligence

During the preparation of this manuscript, the authors used gpt-oss-20b (OpenAI) and Claude Opus 5 to improve grammar, spelling, punctuation, and readability. The authors reviewed and edited the output and took full responsibility for the content of this publication.

## 3. Results

### 3.1. Subjects

**Figure 3:**
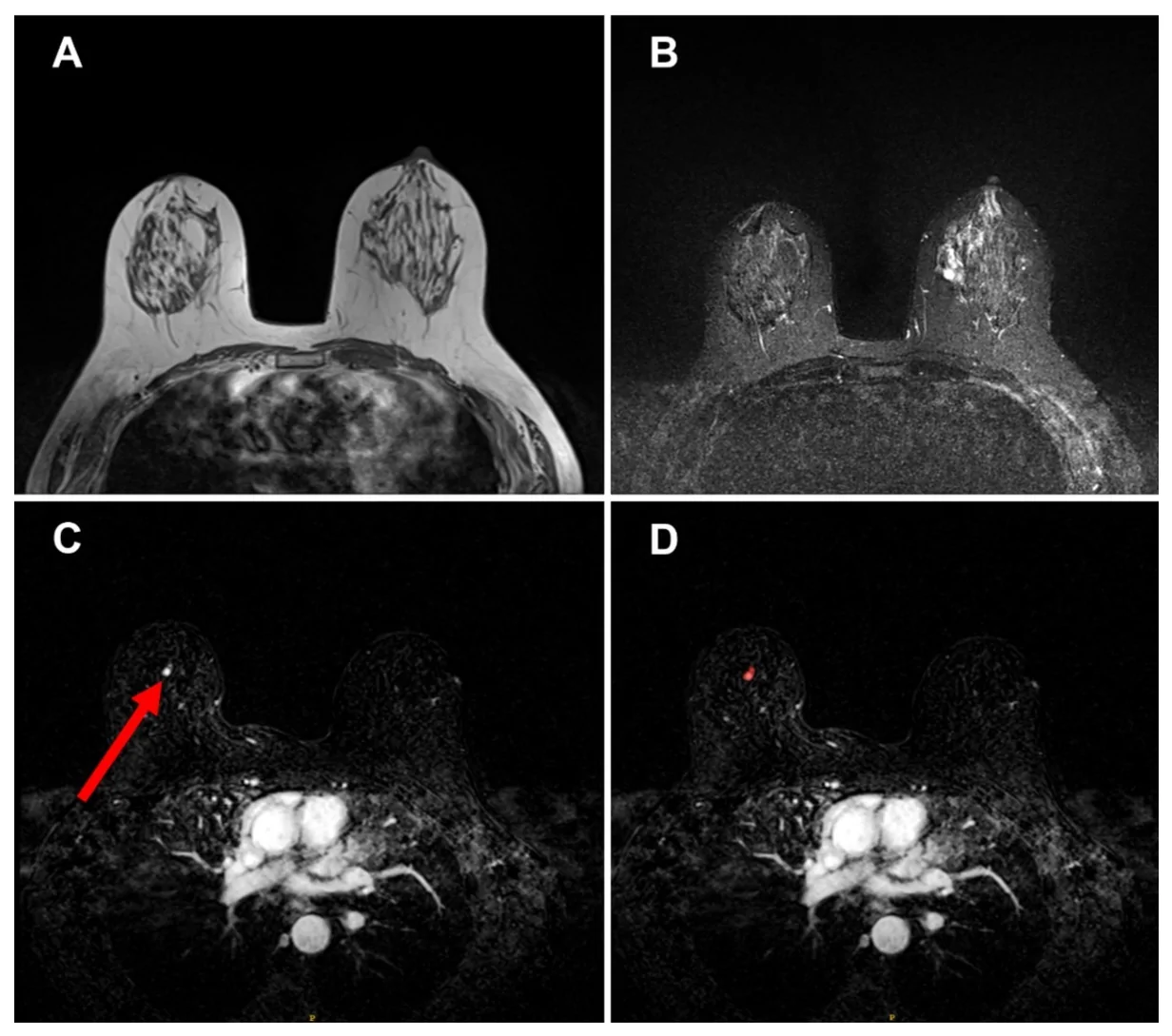
Breast MRI demonstrates focal enhancement. (**A**) Axial T1-weighted image; (**B**) axial T2-weighted STIR sequence; (**C**) axial early-phase post-contrast subtraction image with red arrow indicating the small enhancing lesion; (**D**) corresponding subtraction image with the enhancing lesion segmented and highlighted in red.

This study included 31 enhancing breast lesions from 30 patients. One patient contributed two separate enhancing lesions (one benign and one malignant), which were treated as independent units. The mean lesion size was 5.9 mm ranging from 2.6 mm to 10 mm. Benign lesions (*N* = 21) had a mean size of 5.4 ± 1.7 mm (median 5.2 mm), whereas malignant lesions (*N* = 10) had a mean size of 6.9 ± 1.9 mm (median 6.7 mm). A two-sample *t*-test revealed no statistically significant size difference between the two groups (CI [−3, 0]) mm. The benign group comprised seven epithelial proliferative lesions without atypia, two papillary lesions without atypia, one benign fibroepithelial tumors, two non-proliferative fibrocystic/stromal changes, four trauma-related or inflammatory changes, one lesion with atypia/in situ disease, and four other benign lesions (Table 2). The malignant group comprised six invasive ductal carcinomas and four ductal carcinomas in situ (Table 3). We grouped DCIS and IDC as malignant since both show similar early-phase DCE-MRI enhancement patterns. The mean patient age was 53.6 ± 10.8 years (median 52, range 29–71) for benign cases and 59.8 ± 9.5 years (median 61, range 45–79) for malignant cases. Figure 3 illustrates focal enhancement on breast MRI images.

### 3.2. Radiomics

Of the 105 features, only 44 carried non-negligible information, scoring above 0.2 on the aggregated FIS scale, on which the most informative feature scored 1.81. This aggregated scale results from summing the per run Gini importances, each normalized to sum to one, across the repeated RF runs; the 0.2 value is therefore expressed on this scale and not out of 1 (Table 4). Figure 2 shows the relationship between the number of features and resulting GeoMean values. For each feature set size, the boxplots depict the distribution of GeoMean across repetitions, whereas the solid line represents the average performance trend. The results demonstrate a clear trend of improvement in GeoMean as redundant, or less informative, features are removed. With the full 105-feature set, the average GeoMean was relatively low (approximately 0.60–0.65). However, as the number of features was reduced, the performance gradually increased, indicating that the classifier benefited from the elimination of noisy or irrelevant variables. The peak performance was observed when nine features remained, where the average GeoMean reached values close to 0.84. Beyond this point, further feature removal leads to a decline in GeoMean as essential features are discarded. With fewer than seven features, the performance deteriorated significantly, approaching the levels observed with the initial full-feature set.

**Table 4:** Rank of features for which the Random Forest feature score is greater than 0.2 (glcm: Grey Level Co-occurrence Matrix, glszm: Grey Level Size Zone Matrix, glrlm: Grey Level Run Length Matrix, gldm: Grey Level Dependence Matrix, and ngtdm: Neighboring Gray Tone Difference Matrix).

Rank	Radiomics Feature	Feature Importance	Rank	Radiomics Feature	Feature Importance
#1	gldm_DependenceNonUniformity	1.81	#23	gldm_DependenceEntropy	0.36
#2	glszm_SizeZoneNonUniformity	1.28	#24	gldm_LargeDependenceLowGrayLevelEmphasis	0.36
#3	glcm_JointEnergy	1.12	#25	shape_Maximum2DDiameterRow	0.34
#4	shape_MeshVolume	1.1	#26	firstorder_Energy	0.34
#5	shape_SurfaceArea	1.08	#27	shape_Maximum2DDiameterSlice	0.32
#6	glcm_JointEntropy	0.97	#28	gldm_SmallDependenceLowGrayLevelEmphasis	0.32
#7	glcm_MaximumProbability	0.79	#29	glszm_ZoneEntropy	0.3
#8	glrlm_RunLengthNonUniformity	0.75	#30	glcm_SumEntropy	0.29
#9	shape_VoxelVolume	0.7	#31	shape_Elongation	0.27
#10	shape_SurfaceVolumeRatio	0.68	#32	shape_Maximum3DDiameter	0.27
#11	glszm_LowGrayLevelZoneEmphasis	0.56	#33	firstorder_Skewness	0.27
#12	shape_Sphericity	0.48	#34	firstorder_Kurtosis	0.26
#13	glrlm_ShortRunLowGrayLevelEmphasis	0.47	#35	shape_MajorAxisLength	0.26
#14	shape_Maximum2DDiameterColumn	0.47	#36	ngtdm_Busyness	0.25
#15	ngtdm_Coarseness	0.45	#37	glcm_Idn	0.24
#16	shape_Flatness	0.44	#38	glszm_GrayLevelNonUniformity	0.23
#17	shape_LeastAxisLength	0.42	#39	glrlm_LongRunLowGrayLevelEmphasis	0.22
#18	glrlm_LowGrayLevelRunEmphasis	0.42	#40	glszm_ZonePercentage	0.22
#19	glcm_Correlation	0.42	#41	firstorder_10Percentile	0.21
#20	gldm_LowGrayLevelEmphasis	0.4	#42	glszm_SmallAreaLowGrayLevelEmphasis	0.21
#21	shape_MinorAxisLength	0.4	#43	firstorder_Uniformity	0.20
#22	firstorder_TotalEnergy	0.37	#44	glszm_LargeAreaLowGrayLevelEmphasis	0.20

**Figure 4:**
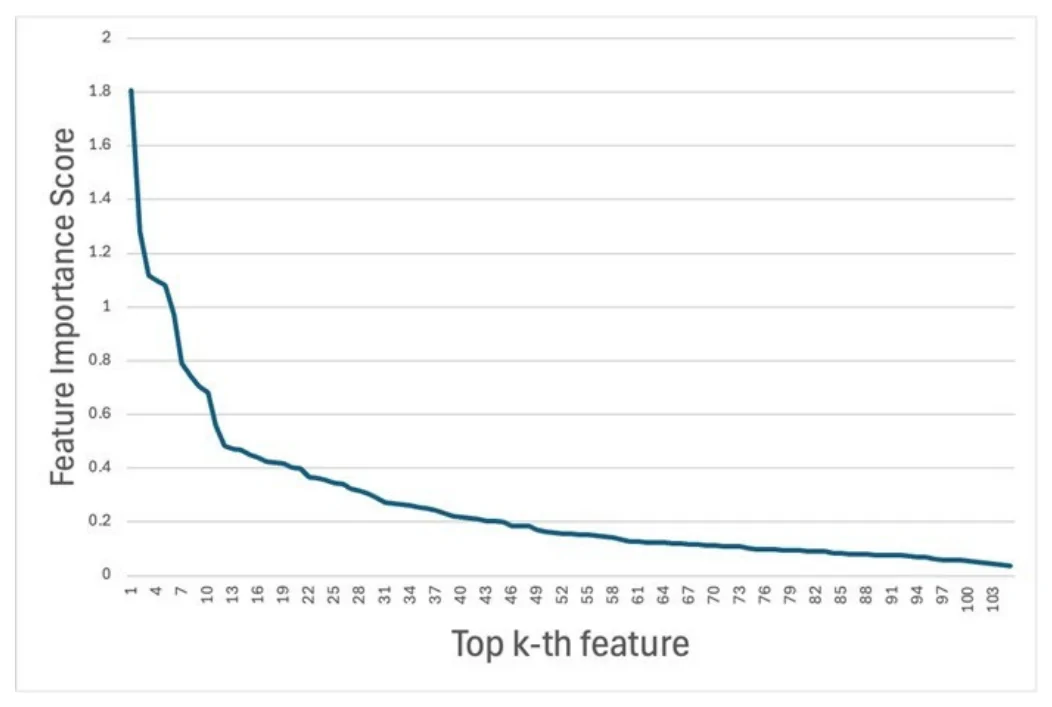
Ranked feature importance scores (FIS) derived from Random Forest feature-ranking. Features are sorted in descending order (rank 1–105).

We also analyzed how the feature importance score varied across the radiomic variables. Figure 4 shows the feature importance scores for a set of radiomic features. The features are ranked in descending order of their importance, with the *x*-axis indicating the position in this ranking, from the 1st (most important) to the 105th (least important).

As observed in many similar classification problems, a small number of top features capture most of the relevant information, whereas subsequent features add diminishing value. This suggests a potential redundancy or lower relevance for lower-ranked features. Up to approximately the 12th feature, the scores drop relatively steeply, indicating a rapid decline in importance among the initial top-tier features. Starting with the 13th feature, however, the scores continue to decrease but at a noticeably slower rate, leveling off more gradually toward near-zero by k = 105. This “elbow” or inflection around k = 9 suggests that the top nine–twelve features are the most critical and including more beyond this point yields minimal additional benefit.

**Figure 5:**
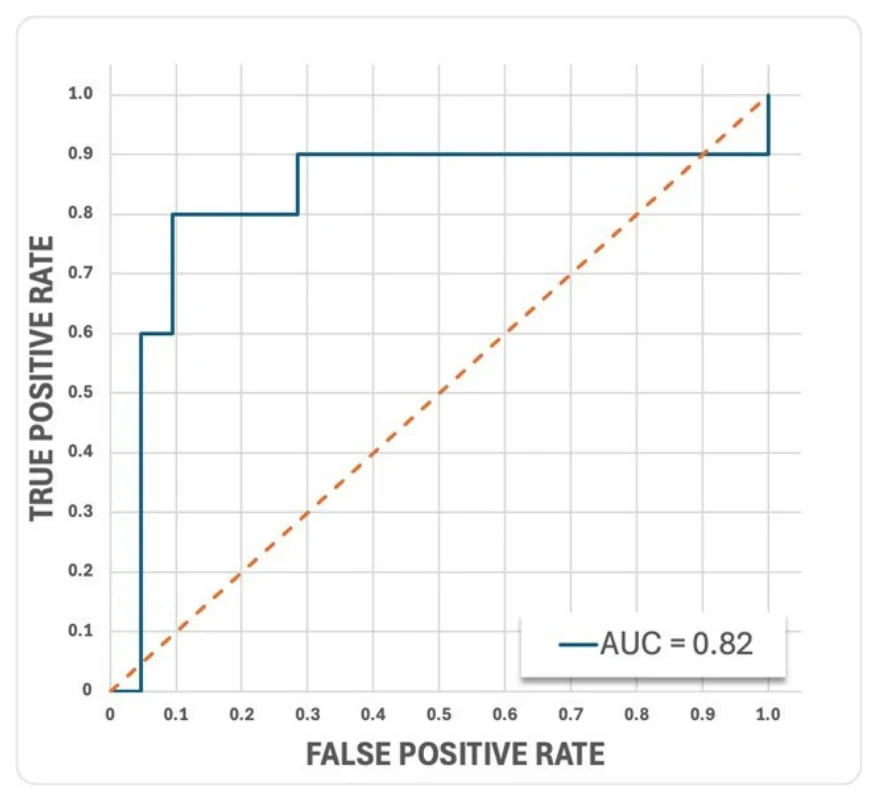
ROC Curve (AUC = 0.82) for the classification of breast cancer subject using the top 9 features (Table 4).

**Figure 6:**
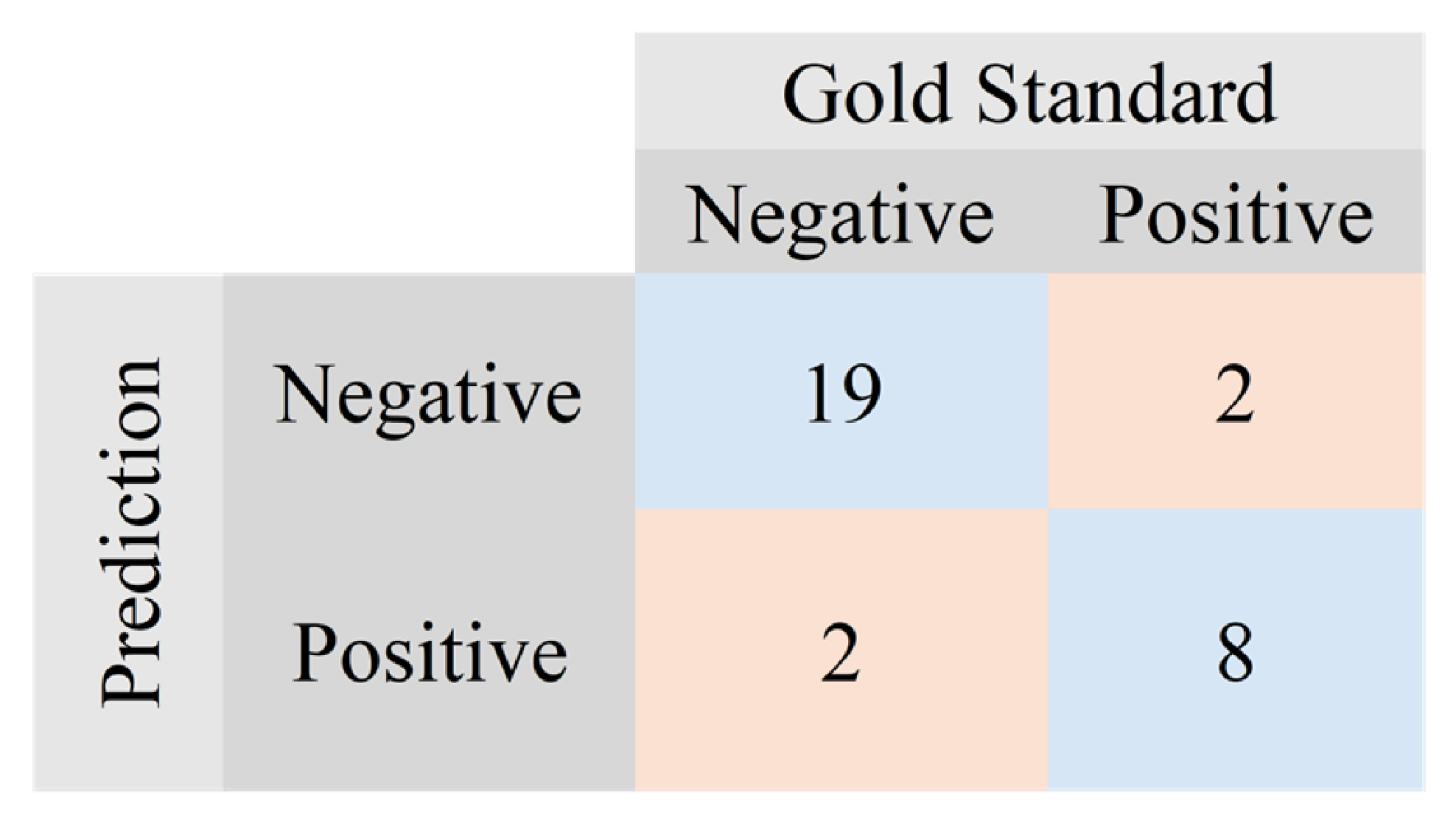
The confusion matrix, which is also observed in the majority of 30-time repeated LOSO-CV experiments.

Using the top nine feature subset, the MLP classifier achieved an AUC of 0.82 (Figure 5). Sensitivity, specificity, accuracy, GeoMean, and the averaged confusion matrix across repetitions are reported in Table 5, while a representative single-repetition confusion matrix is shown in Figure 6. The low standard deviations across all metrics indicate stable classifier performance across subjects despite the subject-independent evaluation imposed by LOSO-CV. Ninety-five percent confidence intervals for sensitivity, specificity, accuracy, and AUC were estimated by a patient-level bootstrap of the out-of-fold predictions, resampling lesions with replacement over 2000 iterations, with predicted probabilities averaged across the repeated runs prior to resampling. The intervals were as follows: sensitivity 0.80 (0.50 to 1.00), specificity 0.89 (0.76 to 1.00), accuracy 0.86 (0.74 to 0.97), GeoMean 0.84 (0.61 to 0.99), and AUC 0.82 (0.53 to 0.95).

## 4. Discussion

In this study, we identified nine radiomic features from sub-centimeter enhancing breast lesions using early-phase DCE subtraction breast MRI images to predict malignancy of small lesions. We developed and trained an MLP algorithm with these features. It achieved a sensitivity of 0.80, specificity of 0.89, accuracy of 0.86, GeoMean of 0.84, and AUC of 0.82 through 30-times repeated LOSO cross-validation. A multilayer perceptron was chosen for its capacity to model nonlinear relationships among radiomic features. We recognize that, given the limited sample size, such a model is more susceptible to overfitting than simpler statistical classifiers. To mitigate this, the input space was reduced to a small feature subset, a compact network architecture was used, early stopping was applied, and class weighting addressed the class imbalance. The resulting model should nonetheless be regarded as exploratory, and comparison against conventional classifiers better suited to small datasets is warranted in future validation.

This repeated evaluation strategy was adopted to account for the sensitivity of MLP classifiers to random weight initialization, thereby minimizing stochastic bias and producing performance estimates that are less dependent on any single random seed. The close agreement between GeoMean and AUC indicates consistent, balanced performance across both classes, and the low standard deviations across all metrics suggest relatively stable classifier behavior despite the subject-independent evaluation imposed by LOSO-CV. Notably, a top-k iterative pruning strategy revealed that focusing on a small subset of top-ranked features substantially improved GeoMean relative to the full 105-feature pool. These findings highlight the importance of feature selection for model generalization and demonstrate that an MLP classifier can be effective when paired with a carefully curated subset of features rather than the entire feature pool.

Among the nine top-ranked features used for classification, six were texture-based, drawn from the GLDM, GLSZM, GLCM, and GLRLM classes, and three were shape-based descriptors (Table 4). The dominance of these mathematically derived texture and morphologic descriptors underscores the value of quantitative “hidden” imaging data in accurate classification of small enhancing breast lesions. The stability of the selected features was evaluated with respect to the RF ranking, which was aggregated over repeated runs with different random seeds. The nine selected features, drawn from complementary texture and shape feature classes, were consistently retained across these repetitions, indicating robustness to the stochasticity of the ranking procedure.

D’Amico et al. similarly applied a radiomics-based machine learning pipeline to distinguish malignant from benign enhancing foci (lesions ≤ 5 mm in maximum diameter) on breast MRI [7]. In their study, radiomic features were extracted from one pre-contrast and four post-contrast timepoints, selected using the TWIST framework, and evaluated using a k-nearest neighbors (kNN) model [7]. Using this workflow, they achieved an AUC of ~0.94 and demonstrated that their most discriminative features were primarily intensity- and texture-based with the highest prediction relevance observed in the early post-contrast phase [7]. When compared with our results, there is meaningful overlap at the feature-family level despite our differences in model choice and imaging timepoint selection. For example, second-order texture descriptors dominated in both studies. A key difference, however, is that D’Amico et al.’s top-performing descriptors also included intensity-based features [7], whereas our top-performing features were dominated by GLCM, GLSZM, GLDM, and GLRLM texture features together and shape descriptors. This observation may reflect differences in cohort compositions as our dataset included lesions up to 10 mm in size. Furthermore, differences in feature extraction and our choice to use phase-1 subtraction images rather than multi-timepoint kinetics may also contribute to varying results.

When expanded to include sub-centimeter enhancing breast lesions, MRI radiomic studies can provide additional context for how “small lesions” behave across different cohorts and workflows. Gibbs et al. evaluated small enhancing BI-RADS 4 using a support vector machine (SVM) model with first-order and texture-based radiomic features, achieving AUCs of 0.75–0.81 [14]. They found that features derived from initial enhancement images provided greater discriminative value than those of later timepoints [14]. Similarly, Lo Gullo et al. applied an SVM-based approach to sub-centimeter enhancing masses in BRCA mutation carriers [15]. They extracted over 100 radiomic features and demonstrated that a small subset of radiomic descriptors combined with clinical data and the first post-contrast scan effectively separated benign from malignant lesions [15]. Although the top-ranked features in both studies differed from ours, their findings collectively reinforce the utility of early post-contrast derived descriptors for radiomic classification and support our decision to focus on early-phase subtraction images for feature extraction.

From a clinical standpoint, incorporating radiomics into a breast radiologist’s workflow might offer the opportunity to leverage supplemental quantitative imaging data to improve the classification of small enhancing breast lesions. A radiomics-based classifier such as the one presented in our study could serve as diagnostic support for small enhancing lesions that are difficult to confidently characterize on morphology alone provided that it is validated in a prospective study with a large cohort. Given that breast MRI is highly sensitive but has suboptimal specificity, an additional quantitative malignancy probability could help radiologists better triage lesions toward short-interval MRI follow-up, targeted second-look via ultrasound, or image-guided biopsy [31,32]. Furthermore, early post-contrast subtraction images are routinely acquired as part of standard breast MRI protocols, posing no additional imaging burden or cost to patients. If validated with larger datasets and paired with automated segmentation, these tools might assist with improving diagnostic consistency [31,32].

This study is not without limitations. First, the small sample size, class imbalance, and single-center design restrict the generalizability of our findings. For small sample size, we utilized LOSO cross-validation strategy, which is a robust validation method for small cohorts. To address our imbalanced dataset, we utilized class weighting within the MLP classifier. Second, we only analyzed first-phase DCE subtraction images, as this is where malignant lesions are most likely to enhance. A dedicated optimization analysis is needed to determine the optimal enhancement phase. Third, this is a retrospective study, and our cohort is not consecutive. Patients were identified based on the presence of a breast MRI followed by a biopsy. Because a biopsy provides the only gold standard, we had to restrict the analysis to those subjects who had tissue sampling. As a result of this, this selection strategy might increase the pre-test probability of malignancy and may overestimate diagnostic performance. Fourth, the reported AUC was computed from the pooled out-of-fold predicted probabilities within each repetition and averaged over thirty repetitions; sensitivity, specificity, accuracy, and GeoMean were obtained at a fixed decision threshold of 0.50 and averaged over the same repetitions. The standard deviations reported across repetitions reflect random seed variability only and do not represent patient-level sampling uncertainty. Patient-level confidence intervals via bootstrap, model calibration assessed by a calibration plot, and decision curve analysis were not performed in this exploratory study and are planned for future confirmatory validation. Given a cohort of 31 lesions with 10 malignant events, the MLP used here is over-parameterized relative to the available sample, and LOO-CV does not fully protect against overfitting when feature and model selection draw on the same data. The reported performance should therefore be interpreted as exploratory. Future confirmatory work should benchmark the MLP against simpler classifiers (penalized logistic regression, linear SVM) using nested, patient-level cross-validation in a larger cohort with external validation before any diagnostic claim is made. Last, feature ranking by Random Forest importance and the selection of the optimal number of features k were performed on the complete dataset prior to the repeated LOSO-CV classification experiments. Because these two model-development steps used all available observations, held-out subjects may have indirectly informed feature ranking and the choice of k. The reported performance metrics should therefore be regarded as internal, potentially optimistic estimates.

We present this as a hypothesis-generating, proof-of-concept study. Given its methodological limitations, specifically, feature selection performed on the full dataset prior to cross-validation, a small sample size, and a lesion-level rather than patient-level analytical framework, the reported performance metrics should not be interpreted as unbiased estimates of generalizability. Future prospective studies are required to validate these findings.

## 5. Conclusions

In conclusion, our preliminary study shows that nine radiomic features extracted from sub-centimeter enhancing breast lesions on early-phase DCE subtraction breast MRI can improve differentiation between benign and malignant lesions when used with an MLP algorithm in a single-center retrospective cohort. These preliminary findings need to be confirmed in future prospective, multicenter studies with larger cohorts to establish clinical utility and reproducibility.

## Figures and Tables

**Table 1:** Dynamic contrast-enhanced (DCE) breast MRI acquisition parameters by field strength (1.5T vs. 3T).

Dynamic Contrast-Enhanced MRI Parameters						
MRI Strength	Repetition Time (TR) (ms)	Echo Time (TE) (ms)	FOV (mm^2^)	Matrix (-)	Slice Thickness (mm)	Flip Angle (°)
3.0T	3.36–5.54	1.24–2.46	220 × 346–400 × 395	224 × 354–416 × 416	0.9–1.3	11–15
1.5T	3.67–4.6	1.42–1.55	341 × 338–422 × 420	384 × 346–480 × 432	1.2–1.5	11–12

**Table 2:** Distribution of benign breast lesion pathology by category and final pathology diagnosis.

Benign Category	Final Pathology Diagnosis	***n***
**Benign** **epithelial proliferative lesions without atypia**	Adenosis with focal usual ductal hyperplasia (UDH)	1
	UDH alone	1
	Columnar cell lesions/change (incl. columnar cell hyperplasia; microcysts/microcalcifications; minute foci of columnar cell change)	3
	Sclerosing adenosis with focal columnar change and microcalcifications	1
	UDH with columnar cell change, adenosis, microcysts, focal perilobular chronic inflammation, and focal pseudoangiomatous stromal hyperplasia (PASH)	1
	**Subtotal**	**7**
**Papillary lesions without atypia**	Sclerosed intraductal papilloma (no atypia)	1
	Sclerosing papilloma with associated UDH and chronic inflammation	1
	**Subtotal**	**2**
**Non-specific benign/limited-sampling findings**	Fragments of benign/normal mammary tissue	3
	No invasive or in situ carcinoma/Negative for metastatic carcinoma	1
	**Subtotal**	**4**
**Trauma-related and inflammatory lesions**	Fat necrosis	2
	Organized fat necrosis with chronic inflammation and coarse calcification	1
	Foreign body-type multinucleated giant cell reaction	1
	**Subtotal**	**4**
**Benign fibroepithelial tumors**	Fibroadenoma	1
	**Subtotal**	1
**Non-proliferative fibrocystic and stromal changes**	Mild stromal sclerosis	1
	Apocrine metaplasia without a dominant mass lesion	1
	**Subtotal**	**2**
**Lesions with atypia/in situ disease**	Lobular carcinoma in situ	1
	**Subtotal**	**1**
	**Total**	**21**

**Table 3:** Distribution of malignant breast lesion pathology by category and final pathology diagnosis.

Malignant Category		***n***
**Invasive Ductal Carcinoma (IDC) Subtypes**	Invasive ductal carcinoma	4
	Infiltrating ductal carcinoma	1
	Invasive ductal carcinoma, poorly differentiated with focal micropapillary features	1
	**Subtotal**	**6**
**Ductal Carcinoma in Situ (DCIS)**	Ductal carcinoma in situ	2
	Apocrine-type ductal carcinoma in situ	1
	Intraductal papilloma, ductal carcinoma in situ	1
	**Subtotal**	**4**
	**Total**	**10**

**Table 5:** Confusion matrix and classification accuracy obtained with the MLP classifier using a 30-time repeated LOSO-CV.

	TP	TN	FP	FN	Sen	Spe	Accuracy	GeoMean
Average	8.0	18.8	2.2	2.0	0.80	0.89	0.86	0.84
StdDev	0.183	0.430	0.430	0.183	0.018	0.021	0.015	0.013
95% CI					0.50–1.00	0.76–1.00	0.74–0.97	0.61–0.99

## Data Availability

The datasets presented in this article are not readily available due to patient privacy and ethical reasons.
